# Alpha-Galactosylceramide/CD1d-Antibody Fusion Proteins Redirect Invariant Natural Killer T Cell Immunity to Solid Tumors and Promote Prolonged Therapeutic Responses

**DOI:** 10.3389/fimmu.2017.01417

**Published:** 2017-11-01

**Authors:** Lianjun Zhang, Alena Donda

**Affiliations:** ^1^Translational Tumor Immunology Group, Ludwig Center for Cancer Research, University of Lausanne, Lausanne, Switzerland; ^2^Department of Fundamental Oncology, University of Lausanne, Lausanne, Switzerland

**Keywords:** bi-functional fusion protein, CD1d-antitumor scFv, NKT cell, DC activation, innate and adaptive immune response, tumor-associated antigen

## Abstract

Major progress in cancer immunotherapies have been obtained by the use of tumor targeting strategies, in particular with the development of bi-functional fusion proteins such as ImmTacs or BiTes, which engage effector T cells for targeted elimination of tumor cells. Given the significance of invariant natural killer T (iNKT) cells in bridging innate and adaptive immunity, we have developed a bi-functional protein composed of the extracellular part of CD1d molecule that was genetically fused to an scFv fragment from high affinity antibodies against HER2 or CEA. Systemic treatments with the CD1d-antitumor fusion proteins loaded with the agonist alpha-galactosylceramide (αGalCer) led to specific iNKT cell activation, resulting in a sustained growth inhibition of established tumors expressing HER2 or CEA, while treatment with the free αGalCer was ineffective. Importantly, we discovered that αGalCer/CD1d-antitumor fusion proteins were able to maintain iNKT cells reactive to multiple re-stimulations in contrast to their anergic state induced after a single injection of free αGalCer. We further demonstrated that the antitumor effects by αGalCer/CD1d-antitumor fusion proteins were largely dependent on the iNKT cell-mediated transactivation of NK cells. Moreover, prolonged antitumor effects could be obtained when combining the CD1d-antitumor fusion protein treatment with a therapeutic peptide/CpG cancer vaccine, which favored the capacity of iNKT cells to transactivate cross-presenting DCs for efficient priming of tumor-specific CD8 T cells. We will also summarize these pre-clinical results with a special focus on the cellular mechanisms underlying iNKT cell unresponsiveness to antigen re-challenge. Finally, we will discuss the perspectives regarding iNKT cell-mediated tumor targeting strategy in cancer immunotherapy.

## Harnessing iNKT Cells for Cancer Immunotherapy

Invariant natural killer T (iNKT) cells represent a unique T cell subset characterized by an invariant TCR alpha chain paired with a restricted number of TCR beta chains both in mouse and humans (1–3). iNKT cells have the capacity to bridge the innate and adaptive immunity (1, 4–6). First, iNKT cells acquire an effector memory phenotype before birth, which allows their trafficking to the site of inflammation where they exhibit direct cytotoxic capacity by the expression of perforin and granzymes. Second, iNKT cells secrete large amounts of effector cytokines very rapidly after activation and are potent activators of NK cells through their fast release of IFNγ. Third, activated iNKT cells communicate with DCs *via* the upregulation of CD40L which promotes DCs licensing and maturation, and subsequently effective CD8 T cell responses (7, 8).

The significance of iNKT cells in antitumor immunity has been well studied in both mouse models and clinics (1, 4, 5, 9–12). Mice lacking iNKT cells are more prone to chemical or p53 loss-induced tumor development (13–15). Along the same line, late-stage cancer patients harbor either decreased numbers of iNKT cells or iNKT cells showing certain functional deficiencies (11, 16–19). Also, head and neck squamous cell carcinoma (HNSCC) patients with lower levels of circulating iNKT cells before radiation therapy show poor 3-year survival as compared to patients harboring higher circulating levels of iNKT cells (20). These observations have triggered the development of iNKT-mediated cancer immunotherapy mainly by the use of the CD1d agonist ligand alpha-galactosylceramide (αGalCer), either as a free drug or loaded on DCs before their adoptive transfer, as reviewed by McEwen-Smith et al. (4) and Robertson et al. (21). These approaches have demonstrated potent iNKT cell activation and subsequent NK cell transactivation and CD8 T cell priming. Despite the potent tumor cytotoxicity and transactivating properties of iNKT cells, clinical responses have remained so far limited, resulting on the one hand from the small numbers of iNKT cells, and on the other hand from their short-lived activation followed by long-term unresponsiveness. To address the issue of the small iNKT cell numbers, the adoptive cell transfer (ACT) of *ex vivo* expanded autologous iNKT cells has been tested in HNSCC and melanoma patients with, respectively, some objective clinical responses and Th1 responses, in particular when iNKT cells were inoculated in the vicinity of the tumor in combination with αGalCer-pulsed DCs (22–24). As mentioned above, the powerful initial αGalCer-mediated activation of iNKT cells is followed by long-term unresponsiveness which is another drawback for the therapeutic manipulation of iNKT cells against cancer (9, 25, 26). In this regard, ACT of αGalCer-pulsed DCs was reported to trigger more effective antitumor immunity than administration of free αGalCer in mouse experimental models and cancer patients (25–28).

More recently, ACT of human iNKT cells transduced with a chimeric antigen receptor (CAR) was reported as a novel and safe platform in a humanized mouse tumor model (29). This attractive approach that requires further validation in immunocompetent hosts would combine the ACT of high numbers of tumor-specific iNKT cells which could be co-activated by αGalCer treatment. However, CAR-T cell immunotherapy represents an expensive personalized cancer treatment and alternative cost-effective treatments would be preferred, such as the development of soluble molecules able to activate and redirect endogenous iNKT cells to the tumor site.

## Tumor Targeting in Cancer Immunotherapies

Major progress in cancer therapy have been obtained by the development of tumor targeting strategies, which mostly involve monoclonal antibodies (mAbs) specific either of tumor-associated antigens (TAA), or soluble factors released by the tumor or inhibitory and activatory receptors expressed by tumor-infiltrating T cells (TILs). For instance, numerous clinical protocols are now routinely involving tumor targeting antibodies such as anti-CD19, anti-HER2, or anti-EGFR combined with chemotherapy or kinase inhibitors for the treatment of, respectively, B cell lymphoma, breast, gastric, and colon cancers (30, 31). In addition to the use of native mAbs, various antibody formats have been developed, which allowed, for instance, the development of a large array of bi-functional molecules by the genetic fusion of an antibody fragment with an effector molecule, such as another antibody fragment, a toxin, a cytokine, or an antigen-presenting molecule. Yet, even a large array of bi-functional proteins have been tested in pre-clinical studies and some clinical trials, very few have so far entered routine clinical application. Among the few bi-functional molecules that are currently under clinical testing, the most promising are the Bi-specific T cell engagers or BiTes, which directly activate T cells against tumor cells by combining an anti-CD3 scFv fragment with another scFv specific of an antigen over-expressed on tumor cells (32). The second class of bi-functional molecules that are currently tested in metastatic melanoma patients are the so-called ImmTACs for “immune mobilizing monoclonal TCRs against cancer,” which combines an optimized TCR specific of HLA-A2/gp100 (IMCgp100) fused to an anti-CD3 scFv (33). While the use of BiTes is restricted to surface-expressed tumor antigens, ImmTACs have the potential to target endogenously processed antigens loaded on MHC I molecules, which greatly increases the possible applications. However, ImmTACs require TCR optimization in the context of defined HLA haplotypes, which represents a personalized and expensive approach. By contrast, BiTes have the advantage to be one drug which fits all patients. Along the same line, we and others have initially developed bi-functional molecules, which combine an MHC I molecule with an antitumor antibody fragment (34–37). We could demonstrate the capacity of these bi-functional molecules to redirect tumor-specific T cells to the tumor site, which led to a significant inhibition of tumor growth (34, 35). More recently, we developed CD1d-antitumor fusion proteins, which offered two main advantages. First, CD1d bi-functional molecules are exploiting a monomorphic antigen-presenting molecule that would fit all patients. Second, when loaded with αGalCer, these CD1d-antitumor fusion proteins will specifically activate Type 1 iNKT cells and redirect both the innate and the adaptive antitumor responses to the tumor site, in view of the transactivating properties of iNKT cells.

## CD1d-Antitumor Fusion Proteins

**Figure 1:**
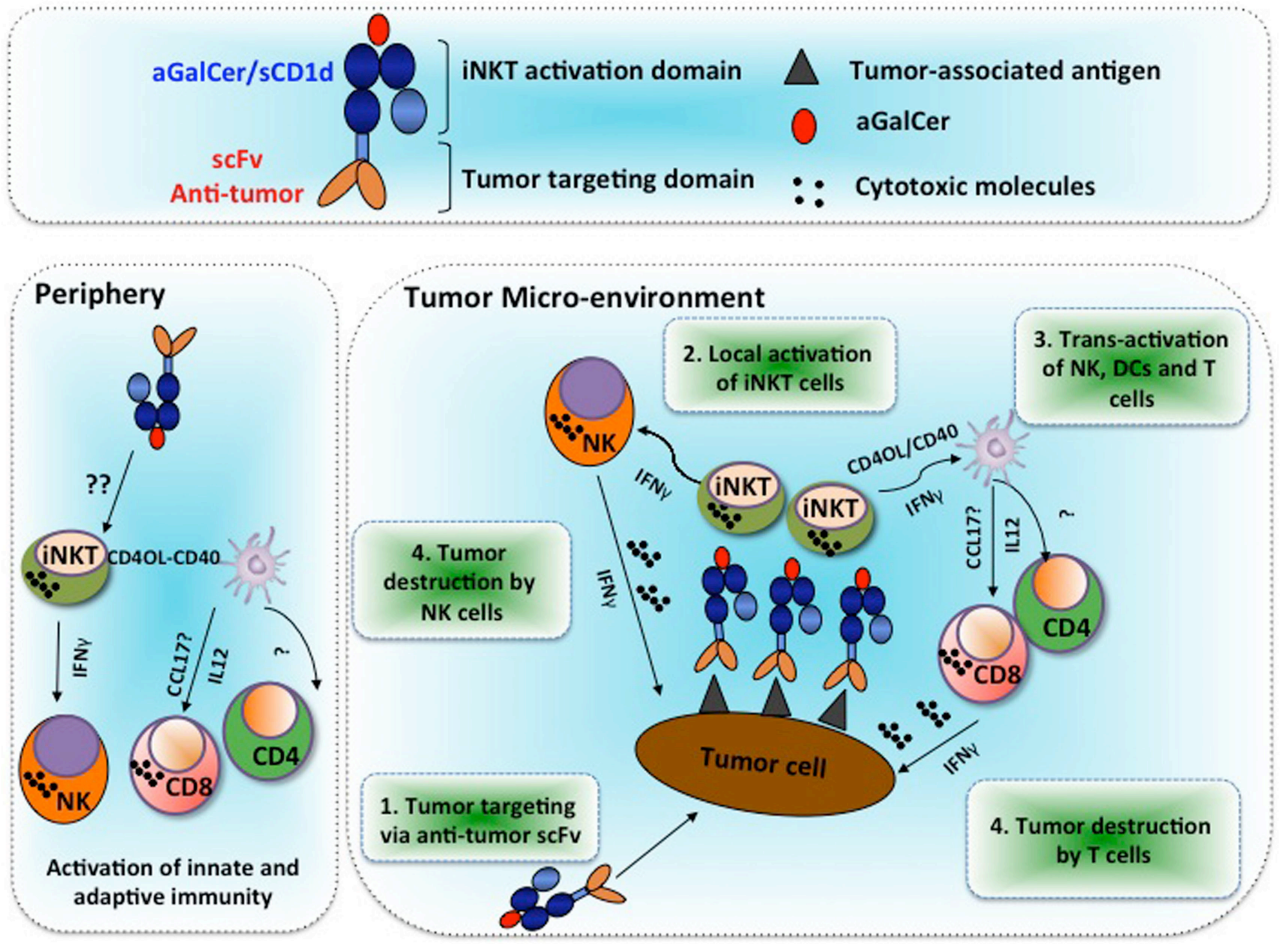
Alpha-galactosylceramide (αGalCer)/CD1d-antibody fusion proteins redirect invariant natural killer T (iNKT), NK, and T cell immunity to solid tumors and promote prolonged therapeutic responses. Tumor microenvironment: Step 1: systemic treatments with the αGalCer/CD1d-antitumor fusion protein lead to its binding to the tumor-associated antigen at the tumor site. Step 2: tumor-bound αGalCer/CD1d-antitumor fusion proteins lead to specific activation of iNKT cells. Step 3: rapid iNKT-mediated transactivation of NK cells and DCs, which then activate and recruit T cells. Step 4: release of IFNγ and cytotoxic molecules from NK and CD8 T cells that mediate targeted tumor destruction. Periphery: soluble αGalCer/CD1d fused or not to an antitumor scFv antibody fragment can also activate iNKT cells in peripheral lymphoid and non-lymphoid organs. The underlying mechanisms for this extra-tumoral activation of iNKT cells remain unclear.

In order to redirect iNKT cell immunity at the tumor site, we have developed CD1d molecules genetically fused to an antibody scFv fragment specific of the HER2 or CEA antigens, which are overexpressed in several cancers (9, 10). Briefly, mouse β2-microglobulin coding sequence (β2M) was fused to the soluble part of CD1d followed by the antibody scFv fragment and a 6xHIS-tag for purification. Flexible glycine/serine linkers were inserted after β2M, CD1d, and scFv to facilitate proper folding. Recombinant proteins were produced in 293-EBNA cells. Strikingly, unlike MHC I/peptide monomer and conventional CD8 T cells, αGalCer/CD1d monomers were able to activate iNKT cells *in vivo* as seen by iNKT TCR down-modulation, as well as iNKT and NK cell proliferation and DC maturation (9). The iNKT cell activation by CD1d monomers may result from the significantly higher binding affinity of the iNKT TCR for αGalCer/CD1d (*K*_D_ ~ 0.3 μM) (38), as compared to conventional TCR for MHC/peptide (*K*_D_ range 1–50 µM) (39). Moreover, it is possible that *in vivo* aggregation or loose cell binding may also facilitate iNKT cell monomer activation. Nonetheless, significant antitumor activity only occurred when the CD1d protein was targeted to a tumor antigen by its fusion to an antibody scFv fragment. First, we demonstrated that B16-HER2 tumor cells pre-coated with αGalCer/CD1d-anti-HER2 fusion proteins totally abolished their potency to initiate tumors (9). In view of these encouraging results, we tested the therapeutic efficacy of αGalCer/CD1d-anti-HER2 proteins in mice bearing established B16-HER2 lung tumor nodules or subcutaneous tumors. In both models, we could demonstrate a significant inhibition of tumor growth, which was dependent on the presence of iNKT and NK cells as the antitumor effects were abolished in CD1d-deficient mice or upon depletion of NK cells (9). The analysis of peripheral lymphoid organs and tumor tissue revealed (i) localization of CD1d-antitumor proteins at the tumor site, (ii) recruitment of iNKT, NK, and T cells at the tumor, (iii) sustained activation of iNKT cells, and (iv) adjuvant effect on CD8 T cell priming (as depicted in the Figure 1).

### Localization of CD1d-Antitumor Proteins at the Tumor Site

CD1d-antitumor proteins were initially validated *in vitro* for their specific binding to tumor cells expressing the relevant tumor antigen. Next, we investigated whether intravenously injected fusion proteins would reach the tumor site in sufficient amounts to attract iNKT cells. Indeed, when injecting radiolabeled αGalCer/CD1d-anti-HER2 proteins in mice bearing on each flank either HER2-positive or HER2-negative tumors, up to twofold more radioactivity was found in HER2-positive tumors, as compared to HER2-negative tumors, while non-targeted αGalCer/CD1d protein did not localize preferentially to any of the tumors, although it induced systemic iNKT cell activation to some extent (9) (see Figure 1).

### Recruitment of iNKT, NK, and T Cells at the Tumor Site

When BrdU-positive iNKT cells in lung tumor nodules, there was a fivefold or twofold increase when compared to untreated mice or the ones treated with untargeted αGalCer/CD1d protein, respectively. Most importantly, we observed a sevenfold enrichment of BrdU-positive NK cells and conventional T cells at tumor site, illustrating that the iNKT-mediated transactivation could trigger their increased proliferation capacity. Interestingly, all three lymphocyte populations were instead decreased in the blood and spleen of CD1d-anti-HER2-treated animals as compared to the untargeted CD1d treatment, which might reflect their preferential recruitment to the tumor site upon HER2 targeting.

### Sustained Activation of iNKT Cells

Strikingly, iNKT cells remained reactive even after multiple treatments with αGalCer/CD1d-antitumor fusion proteins, in contrast to the hyporesponsive state that typically follows the injection of the free ligand αGalCer. This preservation of iNKT cell responsiveness allowed multiple injections of the fusion proteins, which greatly enhanced antitumor efficacy of tumor-targeted CD1d as compared to αGalCer/CD1d molecules targeted to an irrelevant tumor antigen (10). Although iNKT cells remained substantially reactive to several injections of αGalCer/CD1d fusion proteins, we did observe a progressive loss of iNKT cell activation with reduced cytokine production. It is highly possible that a proportion of loaded glycolipid analog was lost from the fusion protein *in vivo* and, thus, processed by APCs, whereby they progressively induced iNKT cell anergy. In this regard, studies are in progress to assess the activities of CD1d fusion proteins loaded with photo-reactive αGalCer analogs that can be UV-crosslinked to CD1d. Initial studies show that complexes of mCD1d with a covalently bound αGalCer are resistant to dissociation and are potent iNKT cell activators *in vitro* and *in vivo* (personal communication, S. Porcelli, Albert Einstein College of Medicine, NY, USA). The validation of these covalently bound αGalCer on CD1d antitumor fusion proteins is in progress with regard to their antitumor activity and capacity to maintain iNKT cells reactive to multiple stimulations.

The mechanism by which systemic treatments with αGalCer/CD1d fusion proteins activate iNKT cells without inducing anergy, as compared to free αGalCer analogs, remains an area of active exploration. High level of surface PD-1 expression has been well defined in exhausted CD8 T cells during chronic viral infection or tumor exposure, which closely correlated with T cell functional decline (40). Likewise, PD-1 expression was also proposed to regulate iNKT cell anergy induction (41, 42). Indeed, upregulation of PD-1 was observed shortly after αGalCer injections, which could last for at least 1 month (41). Moreover, two studies showed that blocking of the interaction between PD-1 and its ligand PD ligand 1 (PD-L1) or PD-L2 at the time of αGalCer injection could prevent the anergy induction of iNKT cells (41, 42). In addition, injection of αGalCer into PD-1 deficient mice failed to induce iNKT cell anergy (42). In a different context, lymphocyte activation gene 3 (LAG-3), another co-inhibitory molecule, was highly expressed on iNKT and NK cells rather than conventional T cells from chronically HIV-infected patients. Interestingly, LAG-3, but not PD-1, was associated with the reduced IFNγ production from iNKT cells, indicating that distinct mechanisms underlying the anergy induction of iNKT cells are context dependent (43). Yet, other mechanisms were also described (44, 45). For instance, deficiency of tuberous sclerosis 1 (TSC1), the upstream inhibitor of mTORC1 signaling, in iNKT cells results in increased resistance to αGalCer induced anergy, which is correlated with impaired upregulation of Egr2 and Grail (46). Altogether, it appears that PD-1 upregulation alone is not enough to mediate iNKT cell anergy.

Interestingly, a recent report showed that, instead of being anergic, iNKT cells were rather reprogrammed toward a suppressive phenotype with the secretion of IL-10 associated with markedly reduced production of effector cytokines (47). Importantly, the study by Wingender et al. (48) showed that Th2-biased αGalCer analogs, which are less hydrophobic than Th1 analogs, are mostly surface-loaded as monomers on CD1d, resulting in a fast and transient iNKT cell activation which preserved their responsiveness to antigen re-challenge. By contrast, the more hydrophobic so-called Th1 αGalCer analogs characterized by a higher critical micelle concentration (CMC) are mostly loaded as micelles and internalized and processed on CD1d *via* the endosomal pathway, leading to a delayed and prolonged iNKT cell activation followed by long-term unresponsiveness (49, 50).

Therefore, we speculate that the surface loading of Th2 analogs on APCs is similar to the loading of αGalCer on CD1d fusion protein which in both cases triggers the fast and transient kinetic of iNKT cell activation, which might be instrumental for their retained reactivity to antigen re-challenge.

### Adjuvant Effect on CD8 T Cell Priming

CD1d-restricted iNKT cells have been shown to promote the transactivation of DCs *via* the CD40L–CD40 interaction (see Figure 1), and their adjuvant properties on the adaptive immunity are well reported (51). For instance, DCs receive cognate “licensing” either from helper T cells or iNKT cells. With regard to iNKT cell, their licensing of cross-priming CD8α^+^ DCs induces them to produce CCL17, which thus attracts CCR4 expressing CD8 T cells (8). However, it remains largely undefined regarding how NK and T cells are recruited upon iNKT cell activation to the tumor site. In this regard, several laboratories have developed vaccine strategies involving either the development of novel αGalCer analogs (52), αGalCer-loaded DC vaccines (53) or DC-targeted nanoparticles loaded with αGalCer (54). With regard to αGalCer/CD1d proteins, repeated iNKT cell activation by untargeted αGalCer/CD1d monomers efficiently promoted the maturation of pro-inflammatory DCs, while αGalCer as a free drug had only a marginal effect (9). Moreover, when tumor-bearing mice received an OVA peptide/CpG-ODN vaccination combined with systemic treatments of αGalCer/CD1d-antitumor fusion proteins, a synergistic expansion of OVA-specific CD8 T cells and NK cells was obtained, as compared to each regimen alone (12). The optimal adjuvant effect on innate and adaptive immune responses likely resulted from the enhanced production of the pro-inflammatory cytokine IL-12 by mature DCs, which was 10-fold higher with the combined stimuli of CpG-ODN and αGalCer/CD1d-anti-HER2 fusion. Most importantly, the combined treatment resulted in an improved enrichment of tumor antigen-specific CD8 T cells and NK cells at the tumor site, associated with better tumor inhibition against tumors co-expressing HER2 and OVA (12). Interestingly, the antibody-mediated depletion of either NK cells or CD8 T cells, demonstrated an early and transient NK-mediated antitumor activity that was quickly replaced by the CD8 antitumor response. Thus, in this context, the direct antitumor activity of iNKT cells was minimal but was instead instrumental for the development of a tumor-targeted innate and adaptive antitumor responses.

## Perspectives

CD1d-antitumor fusion proteins represent an attractive tool to redirect at the tumor site the immunoregulatory properties of iNKT cells on the innate and adaptive immune responses. Importantly, this strategy holds the advantage to maintain iNKT cells reactive to multiple treatments in contrast to the use of an αGalCer analog as a free drug. Yet, the development of covalently bound αGalCer on CD1d fusion proteins will improve their stability *in vivo* and should greatly increase the sustained activation of iNKT cells and antitumor efficacy. Although, these molecules did not show significant liver toxicity, more in-depth pharmacological studies need to be done. Moreover, imaging techniques will help demonstrate that oligomerization of CD1d-antitumor proteins on the tumor cell likely optimize the formation of an immunological synapse with the iNKT cell. However, in view of the low numbers of iNKT cells in cancer patients, the benefit would primarily result from their adjuvant effects rather than their direct antitumor cytotoxicity, unless iNKT ACT is included. Finally, CD1d is monomorphic and a single fusion protein would fit all patients, in contrast to other approaches such as ImmTacs, which involves individual TCRs.

## Ethics Statement

All animal experiments were conducted under an authorization delivered by the Swiss veterinary department.

## Author Contributions

AD has initiated the project and led its subsequent developments. LZ has contributed to the project in a later stage. AD and LZ wrote the review together.

## Conflict of Interest Statement

The authors declare that the research was conducted in the absence of any commercial or financial relationships that could be construed as a potential conflict of interest.
